# Coupling observational methods and the DayCent model to improve examinations of N_2_O production pathways

**DOI:** 10.1007/s10705-026-10504-1

**Published:** 2026-05-20

**Authors:** Shauna-kay Rainford, Julius C. Havsteen, Joachim Mohn, Christof Ammann, Sonja G. Keel

**Affiliations:** 1 Climate and Agriculture Group, Agroscope, Zurich, Switzerland; 2 Laboratory for Air Pollution / Environmental Technology, Empa, Dübendorf, Switzerland

**Keywords:** DayCent, Nitrous oxide emissions, Isotopologues, Water-filled pore space

## Abstract

The traditional calibration approach for process-based models, such as DayCent, consists of the iterative adjustment of model parameters and comparison of the simulated total N_2_O flux to measured observations. However, the contributions of individual production pathways, namely nitrification and denitrification, are uncertain. Here, N_2_O emissions from the soil of sugar beet plots with control (Null) and mineral (NPK) fertilizer treatments were measured by a static chamber technique. The isotopic composition of emitted N_2_O was analyzed to identify the N_2_O production pathways. The latter showed that denitrification was the predominant source of N_2_O emissions at this site. The model’s default settings strongly overestimated the contributions of nitrification. This incorrect allocation of N_2_O emissions to nitrification could partly be explained by the model’s tendency to underestimate the soil water content during the growing season. DayCent model parameters were also manually adjusted to better represent the observation derived contributions of nitrification and denitrification. Although, this “expert-informed approach”, showed a slightly lower performance concerning the cumulative N_2_O flux (Null: RMSE = 0.37 kg N ha^−1^ yr^−1^, NPK: RMSE = 0.50 kg N ha^−1^ yr^−1^) than the traditional calibration (Null: RMSE = 0.15 kg N ha^−1^ yr^−1^, NPK: RMSE = 0.10 kg N ha^−1^ yr^−1^), it may be considered as more representative because it better reflected the higher contribution from denitrification shown by the isotope data. This study demonstrates that the inclusion of observational methods, such as isotope measurements, can provide important insight into model function and improve pathway-specific estimation of N_2_O emissions in DayCent.

**Supplementary Information:**

The online version contains supplementary material available at 10.1007/s10705-026-10504-1.

## Introduction

Nitrous oxide (N_2_O) is a potent greenhouse gas (GHG) that contributes to global warming and to the depletion of the stratospheric ozone layer (Montzka et al. 2011; Ravishankara et al. 2009; WMO 2022). The agricultural sector represents the largest source of anthropogenic N_2_O emissions (3.6 Tg N yr^−1^, or ~ 56%) due to its use of nitrogen (N) fertilizer and the application of slurry and manure in croplands (Lu et al. 2022; Tian et al. 2024). Without abatement, N_2_O emissions will exceed predicted levels under all scenarios in the Coupled Model Intercomparison Project Phase 6 (CMIP6) of the Intergovernmental Panel on Climate Change (IPCC 2021; Gidden et al. 2019; Tian et al. 2020). Many studies have suggested that quantifying the contributions of N_2_O emissions from the microbial-mediated processes of nitrification (the oxidation of ammonium to nitrate) and denitrification (the reduction of nitrate via N_2_O to dinitrogen) can aid in the identification of emission hotspots and the implementation of targeted mitigation strategies (Cui et al. 2024; Grados et al. 2022; Hénault et al. 2012; Pan et al. 2022). However, the contributions of N_2_O emissions from each process remain a large source of uncertainty due to their simultaneous occurrence in soil (Sihi et al. 2020). It is therefore essential that we improve our ability to allocate N_2_O emissions accurately to the two key production pathways.

The use of process-based models, such as DayCent (the daily time-step version of the Century model), is a widely established bottom-up approach to estimate N_2_O emissions from arable soil across a range of spatial scales (Tang et al. 2024). The N-gas sub-model within DayCent quantifies N_2_O emissions by examining key ecosystem factors that influence soil N_2_O flux (Del Grosso et al. 2000; Parton et al. 2001). Similar to other process-based models, N_2_O emissions from nitrification and denitrification within DayCent are regulated by soil water content, soil oxygen status, and temperature (Heinen 2006; Wang et al. 2023). The accuracy of DayCent’s predictions of N_2_O emissions via these pathways is dependent on the model’s ability to replicate soil conditions in the field (Brilli et al. 2017; Guest et al. 2017; Yang et al. 2017).

Several studies have shown that DayCent’s ability to estimate N_2_O emissions can be improved through the calibration of model parameters that control the flow of N as it is transformed through the nitrification and denitrification pathways (dos Reis Martins et al. 2022; Necpalova et al. 2018). The traditional calibration approach entails the user manually adjusting individual model parameters in an iterative fashion. DayCent can also be coupled with advanced inverse modeling tools that accelerate the identification of parameter effects on model outputs (dos Reis Martins et al. 2022; Necpalova et al. 2015; Rafique et al. 2015). Both techniques use comparisons of the simulated total N_2_O flux with measured observations to refine the model. While these approaches improve estimations of N_2_O emissions, the contributions from nitrification and denitrification are still poorly understood (Berardi et al. 2020; Dueri et al. 2023). This is due in part to the methodological difficulties involved in measuring the gaseous and soluble components of the nitrification and denitrification processes, which hinder direct comparisons to simulated N-gas production and emissions within process-based models (Friedl et al. 2020; Groffman et al. 2006).

Parameter values within DayCent can also be adjusted using information derived from observational methods, which provide important insights into both ecosystem and model function (Del Grosso et al. 2020). For example, soil water-filled pore space (WFPS) is the characterization of the proportion of soil pore space that is filled with water and is a useful measure that denotes soil water content and the availability of oxygen throughout the soil profile (Linn and Doran 1984). Many studies have shown that soil water content is a key regulator of N_2_O emissions, because it directly affects microbial activity and thus microbially mediated N transformation processes (Kuang et al. 2019; Liu et al. 2022; Wang et al. 2023). Given the importance of soil water content for N_2_O production from nitrification and denitrification, this metric can be used to identify discrepancies between the simulated and the observed soil moisture environment on the field that may impact N_2_O emission via each pathway (Bateman and Baggs 2005).

Isotopic methods have been applied in numerous studies to evaluate the contribution of individual N_2_O source processes and can be combined with process-based models to constrain model parameters. In this regard, singly substituted isotopologues of N_2_O (^14^N^15^N^16^O, ^15^N^14^N^16^O, ^14^N^14^N^18^O) become critically important. These species differ from the most abundant N_2_O isotopologue (^14^N^14^N^16^O) by the substitution of one nitrogen atom at the central (α) or terminal (β) nitrogen position by ^15^N, or the oxygen atom by the rare ^18^O isotope (Toyoda and Yoshida 1999). Their relative abundances are expressed using δ-notation, where δ^15^N = (R(^15^N/^14^N)_sample_/R(^15^N/^14^N)_reference_)-1 denotes the relative difference in isotope ratio of the sample versus a reference material in per mil (‰). By extension, position-specific δ-values are defined as follows: δ^15^N^α^ for ^14^N^15^N^16^O/^14^N^14^N^16^O, δ^15^N^β^ for ^15^N^14^N^16^O/^14^N^14^N^16^O. The term site preference (SP) of ^15^N (δ^15^N^SP^ = δ^15^N^α^-δ^15^N^β^) describes the preference of ^15^N substitution in the central α-position as compared to the terminal β-position within the N_2_O molecule. Likewise, bulk δ^15^N (δ^15^N^bulk^ = (δ^15^N^α^ + δ^15^N^β^)/2) represents the average N isotopic composition of the N_2_O molecule. In its simplest terms, the parameter δ^15^N^SP^ offers a distinctive fingerprint independent of the isotopic composition of the substrates, with higher values (~ + 33 to + ⁠38 ‰) typically indicating nitrification and lower values (~ -5 to + ⁠5 ‰) commonly associated with the denitrifying pathways (Yu et al. 2020). For interpretive clarity, and because DayCent only discriminates between nitrification and denitrification, we use this field-informed and simplified categorization, while noting that in natural systems multiple N_2_O production pathways with partially overlapping isotopic signatures can occur. For example, high δ^15^N^SP^, often attributed to nitrification, can also be consistent with fungal denitrification, while δ^15^N^SP^ around 0 ‰, in principle, can reflect either bacterial denitrification or nitrifier denitrification (Yu et al. 2020). By comparison, δ^15^N^bulk^ reflects the integrated isotope signature of the N substrate pool and fractionation processes during N_2_O production and reduction, with broad, overlapping ranges in δ^15^N^bulk^ possible for both nitrification and denitrification dominated emissions. In parallel, δ^18^O traces the origin of the O atom, e.g., from soil water, oxidized N substrates or molecular O_2_, and exchange processes, further aiding in the distinction between pathways. Isotopic information thus provides important pathway-specific fingerprints that enable improved source attributions of N_2_O, thereby reducing uncertainties and enhancing predictions in model estimates (Yu et al. 2020). However, the relationship between WFPS and N_2_O production is not constant across soils because it is governed by soil physical controls on aeration, including gas diffusivity, which is influenced by soil parameters such as texture and bulk density, and by oxygen consumption rates linked to degradable organic matter. Results should therefore be interpreted in conjunction with these field-specific constraints (Balaine et al. 2016).

While the use of isotope analyses to constrain model parameters is not a traditional form of model “calibration,” the use of observational data and process-based understanding can be used to compensate for inaccurate representations of processes within DayCent (Del Grosso 2020). An early application of this combined approach coupled an N isotope model with DayCent to constrain model parameters for the estimation of gaseous N emissions along an ecological (soil and precipitation) gradient (Bai and Houlton 2009). More recently, isotopic composition of N_2_O measurements were paired with the stable isotope model SIMONE and used to parameterize and test the process-based model LandscapeDNDC in its identification of N_2_O source processes (Denk et al. 2019; Ibraim et al. 2020).

Refining process-based understanding of nitrification and denitrification in biogeochemical models such as DayCent is crucial to improving estimations of N_2_O emissions in agricultural soils. Therefore, the objectives of this study were to determine the primary source of N_2_O emissions at the field scale based on isotopic analysis, to identify potential discrepancies between the DayCent model and the soil water environment at the site, and to evaluate the performance of DayCent to estimate N_2_O emissions from nitrification and denitrification through different model parameterizations.

## Materials and methods

### Site description

The field component of this study was conducted at the Demo87 (Demo) trial, which is located at the Agroscope-Reckenholz Research Station in Zürich, Switzerland (47° 25′ 31″ N, 8° 30′ 59″ E; 443 m asl). This long-term fertilization experiment assesses the effect of nutrient deficiencies on the development of different summer and winter crops. Mean annual temperature at the site is 9.4 °C and mean annual precipitation is 1,031 mm. The topsoil is classified as an Eutric Cambisol (WRB), and it has a loam soil texture (20% clay, 33% silt, 47% sand), and an organic carbon content of 3%.

A detailed description of the management practices at the Demo site can be found in Frei et al. (2024). Briefly, the demonstration trial was established in 1989 on a managed meadow (~ 0.7 ha). The trial consists of seven-year crop rotations that are arranged in a non-replicated staggered-start design (Loughin 2006) with eight different mineral and organic fertilizer treatment plots (5 × 8 m), each. The seven-year crop rotation includes spring wheat (*Triticum aestivum L.*), sugar beet (*Beta vulgaris L.*), silage maize, potato (*Solanum tuberosum L.*), winter barley (*Hordeum vulgare L.*), and two consecutive years of grass-clover-ley, (main species: *Trifolium pratense L.*, *Trifolium repens L.*, *Dactylis glomerata L.*, *Festuca pratensis Huds.*, *Lolium perenne L.*, *Phleum pratense L.*). However, the crop rotation experienced a deviation in which sugar beet, instead of spring wheat, was planted after the second year of grass-clover-ley cultivation in 2023. For the present study, measurements were performed in 2024 on the mineral fertilizer (NPK) and the non-fertilized (Null) treatment of the rotation with sugar beet in that year. Grass-clover-ley remained on the plots until they were plowed on 8 April 2024 and harrowed 10 April 2024. Sugar beets were planted on 11 April 2024. The NPK treatment received ammonium sulfate (50 kg N ha^−1^) on 7 May 2024, and ammonium nitrate (50 kg N ha^−1^) on 21 May 2025. Crops were harvested on 10 October 2024.

### Soil water content measurements

Soil volumetric water content (VWC) was measured in 2023 and 2024 using a TEROS 12 soil moisture sensor (METER Group, Pullman, WA, USA) that was installed in the NPK treatment of the sugar beet and winter barley plots at the 5 cm soil depth. The soil VWC was recorded at fifteen-minute intervals, and daily averages were determined for comparisons to the DayCent model. VWC measurements for the sugar beet plot failed for the period between April and June 2024. Therefore, we used the parallel measurements from a neighboring plot (about 50 m distance) planted with barley to fill most of this gap in the time series. VWC measurements from the investigated NPK treatment plot for the year 2023 (during which time grass-clover-ley was cultivated) were also included to compare observed and simulated soil water conditions.

The WFPS was calculated as the quotient between VWC and total porosity (Linn and Doran 1984), in which soil porosity was determined as a function of bulk density (BD) and a soil particle density (PD) of 2.65 g cm^−3^ (Minasny et al. 1999):

1 $$WFPS=100* \frac{VWC}{\left(1- \frac{BD}{PD}\right)}$$

For this purpose, a constant BD value of 1.39 g/cm^3^ was used, which had been determined for the investigated plot in 2021 as an average of four cylinder samples taken for a depth of 5–10 cm.

### Nitrous oxide flux measurements and identification of N_2_O production pathways using isotopologues

Gas fluxes and microbial N_2_O isotopic source signatures were determined with weekly resolution using air samples from an automatic time integrating chamber (ATIC) system. In short, after each chamber closure, the ATIC system automatically collects sequential gas samples into impermeable 5 L gas bags (30,228-U, Supel™-Inert Multi-Layer Foil, Sigma-Aldrich, USA) according to the approach outlined in Wang et al. (2022). For the presented study three chambers were applied on both considered treatments (NPK, Null). Each chamber was closed for 15 min every 4 h. During each closure event, the following timings were applied: four consecutive headspace samples were collected for 15 s, with sampling for Bag1 beginning at 3.50 min after closure, followed by filling of the subsequent bags (Bag2, Bag3, Bag4) at 7.25, 11.50 and 14.25 min, respectively.

Gas concentrations (N_2_O, CH_4_, CO_2_) and singly substituted isotopic composition of N_2_O (δ^15^N^α^, δ^15^N^β^ and δ^18^O) were analyzed using commercial cavity ring-down spectroscopy (CRDS; Picarro Inc., USA). Measurements of CH_4_ and CO_2_ were performed with a G2401-m analyzer (serial number: 2829-CFKADS2266), while N_2_O, δ^15^N^α^, δ^15^N^β^ and δ^18^O measurements were conducted on two G5131-*i* analyzers (serial numbers: 5080-DAS-JDD S5089 and 5056-PPU-JDD S5065) at Empa, Dübendorf, Switzerland. The raw δ-values were corrected for spectral interferences, non-linearity as well as instrumental drift and calibrated by intermittent analysis of reference gases, expressed relative to the atmospheric N_2_ (AIR-N_2_) scale for ^15^N/^14^N and the Vienna Standard Mean Ocean Water (VSMOW) scale for ^18^O/^16^O (Mohn et al. 2022; Ostrom et al. 2018). Gas concentrations were calibrated against the established reference scales, provided by the Global Atmosphere Watch (GAW) program for greenhouse gases from the World Meteorological Organization (WMO). Full details on the correction and calibration approach are provided in Havsteen et al. (2026, under review, Atmos. Meas. Tech.).

This analytical workflow enabled the calculation of weekly mean fluxes of N_2_O, CH_4_ and CO_2_ per chamber using a linear regression approach for gas concentration over time, with an imposed quality criterion of R^2^ ≥ 0.7 for data acceptance. The concentration-to-flux conversion considered dynamic molar volumes, calculated from the mean ambient temperature over the measurement period, recorded two meters above the ground at the Zürich-Affoltern weather station (REH; 47.4277° N, 8.5180° E; 444 m a.s.l.), which is part of the automatic monitoring network operated by MeteoSwiss (WIGOS ID: 0-20000-0-06664). The microbial N_2_O isotopic source signatures were derived using a two-endmember mixing model (Keeling plot) (Keeling 1958), where each gas sample collected from a chamber represents a mixture of ambient atmospheric N_2_O and N_2_O from soil-derived microbial production. To determine the microbial source endmember for a specific time and chamber, isotopologue values were plotted against the reciprocal N_2_O concentrations of the respective bags. The intercept of the regression line provides an estimate of the microbial N_2_O source signature (δ^15^N^α^, δ^15^N^β^ and δ^18^O). To assess the data quality, the regression-derived isotopic composition at ambient N_2_O concentration was compared to actual values. Three quality classes were defined: Quality 1 (Q1) for agreement within 4 ‰, Q2 (4–6 ‰) and Q3 (6–8 ‰). Regression lines with deviations in ambient isotopic signatures from actual values of more than 8 ‰ were excluded from further analysis. If a sample sequence was measured on two G5131-*i* analyzers, duplicate analysis provides two Keeling plot intercepts, which were averaged before further interpretation. To avoid false positive results under low flux scenarios, an additional criterion was applied, requiring a minimum N_2_O flux threshold of 75 µg N_2_O-N m^−2^ h^−1^ combined with a regression quality threshold of R^2^ ≥ 0.7.

### Model description

DayCent is a process-based ecosystem model that simulates carbon, nitrogen, phosphorus, and sulfur dynamics in plant-soil systems (Del Grosso et al. 2000). The main inputs of the DayCent model include soils data (soil texture, bulk density, pH), field management data (crop type, fertilization, tillage, harvest), and daily weather data (minimum and maximum temperature and precipitation).

The N-gas sub-model in DayCent is based on the “leaky pipe” concept (Firestone and Davidson 1989), in which total N gas emissions are proportional to N cycling and soil diffusivity. Within the N-gas sub-model, the nitrification and denitrification processes represent the figurative “pipes” which facilitate the transformation of N, wherein nitrate and dinitrogen are the respective end products of each process. Nitrous oxide emissions from nitrification are a function of the soil ammonium concentration, WFPS, soil temperature, pH, and soil texture. Modeled N_2_O flux from denitrification is driven by the soil nitrate concentration, the heterotrophic CO_2_ respiration rate (which is representative of labile C availability), WFPS, and soil physical properties that impact gas diffusivity (such as soil texture and bulk density). The pipe is said to be “leaky” because N_2_O and nitric oxide (NO) are by-products of nitrification and denitrification and the amount of N_2_O and NO that is produced is determined by soil water content, soil temperature, and soil physical properties that impact gas diffusivity (the size of the holes in the pipe) during intermediate steps.

Within DayCent, N cycling is controlled by several adjustable model parameters (Del Grosso et al. 2000). Sensitivity analyses have found that the calibration of specific, N-cycle related, model parameters improve the model’s ability to simulate N_2_O emissions (Necpalova et al. 2015; Rafique et al. 2013). In 2022, dos Reis Martins et al. published a study that used approximately 24,000 daily N_2_O flux observations from six cropland sites located in Western Europe, including sites in Switzerland. DayCent model parameters were calibrated using the Model-Independent Parameter Estimation (PEST) statistical tool for each site, and model performance was evaluated using a leave-one-out cross-evaluation. This study found that improvements to N_2_O predictions at each site were associated with seven N-cycle related parameters (Table 1) (dos Reis Martins et al. 2022). Here, we focused on the three parameters that influence the effect of soil water content on the nitrification and denitrification processes within the model. Specifically, the N2Oadjust_fc and N2Oadjust_wp parameters, respectively, control the maximum and minimum proportions of nitrified N lost as N_2_O when soil conditions are at field capacity and wilting point. N_2_O emissions are also directly impacted by the wfpsdnitadj parameter, which controls the inflection point for the effect of WFPS on denitrification, wherein values less than one permit denitrification to occur at lower soil water content and values greater than one require wetter conditions for denitrification to occur.

**Table 1:** Three different sets of DayCent parameter values which are relevant for soil nitrogen cycling

DayCent Parameter	Description	Default	Traditional	Expert-informed
Ncoeff	Minimum water/temperature reduction on nitrification	0.030	0.027	0.027
N2Oadjust_fc	Maximum proportion of nitrified N lost as N_2_O at field capacity	0.025	0.000	0.001
N2Oadjust_wp	Minimum proportion of nitrified N lost as N_2_O at wilting point	0.020	0.004	0.001
MaxNitAmt	Maximum daily nitrification amount	1.500	3.690	3.690
Netmn_to_no3	Fraction of new net mineralization that is converted to NO_3_ each day	0.200	0.359	0.359
wfpsdnitadj	Adjustment on inflection point for the water filled pore space effect on denitrification curve	1.000	1.400	1.000
N2N2Oadj	N_2_/N_2_O ratio adjustment coefficient	1.000	1.159	1.000

The value of the default parameters is outlined in Hartman et al. (2018). The optimized parameter values from dos Reis Martins et al. (2022) and the values for the traditional calibration and the expert-informed approaches were used for simulations in DayCent

### Model parameterization and calibration

Model simulations were conducted for the plots with Null and NPK treatments on which sugar beet was cultivated in 2024. Total N_2_O and N_2_O emissions from nitrification and denitrification were simulated in DayCent using the same climate, physical and chemical soil parameters, and management data. Local meteorological factors (minimum and maximum temperature, precipitation, relative humidity, windspeed, and solar radiation) were collected from the REH weather station. For all simulations, soil nutrient pools were initialized to equilibrium by growing an unfertilized grassland system under flooded conditions between 1801 and 1969. This was done in accordance with Wang et al. (2025), as flooded conditions were assumed because the site is now drained. Next, a post-drainage period was simulated to reflect the land-use history between 1970 and 1988. This base simulation included a year of grass-clover-ley followed by a three-year arable crop rotation that was fertilized with manure. In the final period between 1989 and 2024 (i.e., the duration of the Demo Trial), separate simulations were conducted to reflect the cultivation histories (i.e., Null and NPK fertilizer treatments) for the experienced crop rotation of the specific plots on which the measurements in this study were performed. In 2024, mineral fertilizers (50 kg N ha^−1^, ammonium sulfate on 7 May, and 50 kg N ha^−1^, ammonium nitrate on 21 May) were applied to the sugar beet NPK treatment, providing a total of 100 kg N ha^−1^ for the growth of this crop.

For this study, the DayCent parameters characterized by the maximum proportion of nitrified N lost as N_2_O at field capacity (N2Oadjust_fc), the minimum proportion of nitrified N lost as N_2_O at wilting point (N2Oadjust_wp), and the inflection point for the effect of WFPS on denitrification (wfpsdnitadj) were manually calibrated using the traditional and expert-informed approaches, respectively. Both approaches used values of the other N cycle related parameters based on the multi-site calibration by dos Reis Martins et al. (2022) and were performed by adjusting one parameter at a time while keeping the others constant. For the traditional approach, the three parameters were modified iteratively between a range of values, and parameter values were adjusted based on how well the simulated total N_2_O output reflected observations. This classical approach avoided heuristic judgement made on behalf of the user and considers field observations of total N_2_O fluxes. The expert-informed approach assigned specific values for each parameter based on information gathered from the results of the WFPS and the isotopic composition of N_2_O observations. Specifically, the WFPS data from 2023 was used to identify potential discrepancies between the observed and simulated soil moisture content, and the isotopic composition of the soil N_2_O endmember was determined using the Keeling plot approach to identify the primary N_2_O production pathway in the soils at the Demo site. In DayCent, the N2Oadjust_fc and N2Oadjust_wp parameters control the impact of WFPS on N_2_O emissions via nitrification and the wfpsdnitadj parameter directs the soil moisture conditions under which N_2_O emissions via denitrification can occur. Within the model, lower values of the N2Oadjust_fc and N2Oadjust_wp parameters limit the release of N_2_O emissions via nitrification, and the wfpsdnitadj parameter facilitates the occurrence of N_2_O emissions from denitrification under dry or wet soil moisture conditions. Parameter values in the expert-informed approach were selected to compensate for discrepancies in the model’s interpretation of the effect of soil moisture content on N_2_O emissions from nitrification and denitrification at the Demo site.

The simulations were calibrated based on the relationship between the simulated total N_2_O flux and the measured N_2_O flux observations throughout the year. We compared the cumulative total N_2_O flux, as well as the cumulative N_2_O emissions from nitrification and denitrification from DayCent simulations using parameter values from the model’s default settings, the traditional calibration, and the expert-informed approach (Table 1).

### Statistical analysis

Since the observed water-filled pore space dataset was incomplete for 2024, we also included the 2023 data in the WFPS evaluation. The coefficient of determination (r^2^) was calculated to measure how well WFPS measurements collected during 2023 and 2024 were replicated by the model. As the same input data such as soil bulk density was used to determine the WFPS at this site in both years, differences in the simulation of WFPS within DayCent between 2023 and 2024 would result from interannual variability in weather conditions, namely the amount of precipitation that occurs throughout the year (Hartman et al. 2018). To ensure that there were no substantial differences in weather conditions between 2023 and 2024, we evaluated the means of the monthly minimum and maximum temperature and precipitation for each year using the Welch Two Sample t-test.

Model calibration was evaluated by calculating the root mean squared error (RMSE) between the model outputs and the corresponding field measurements using the following equation:

2 $$RMSE=\sqrt{\sum_{i=1}^{n}{({S}_{i}-{O}_{i})}^{2} / n}$$

where O_*i*_ and S_*i*_ represent the observed and simulated values at point in time *i* and *n* denotes the number of observations. All statistical analyses were performed using R version 4.4.2 (R Core Team 2024). P-values less than 0.05 were considered statistically significant.

## Results

### Comparison of soil moisture content

**Fig. 1:**
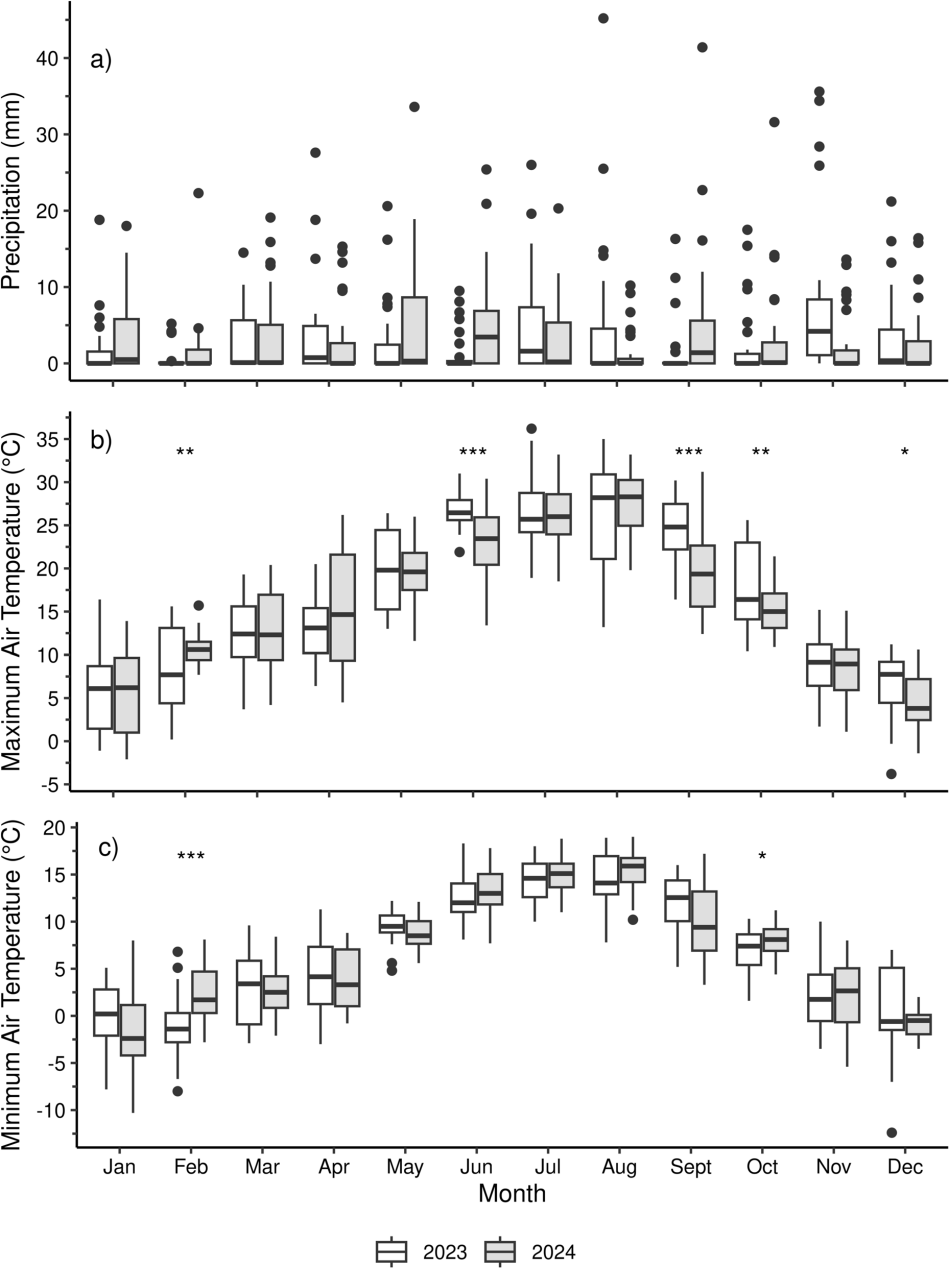
Comparison of the precipitation **a**, maximum air temperature **b**, and minimum air temperature **c** measurements from the Zürich-Affoltern (REH) weather station located near the Demo site in Switzerland. Significant differences, as determined by the Welch Two Sample t-test, between the monthly means for 2023 (white) and 2024 (gray) are indicated by the following levels: *p ≤ 0.05, **p ≤ 0.01, and ***p ≤ 0.001

Due to the strong influence of precipitation on WFPS simulations within the DayCent model, a comparison of weather factors between 2023 and 2024 was conducted to confirm the reliability of extrapolating inferences drawn from WFPS simulations across different years. There were no significant differences in precipitation between 2023 and 2024 (Fig. 1a). However, maximum air temperature was significantly higher in 2023 during June (*p* < 0.001), September (*p* < 0.001), October (*p* < 0.01), and December (*p* < 0.05), and significantly higher in 2024 during February (*p* < 0.01) (Fig. 1b). Similarly, the minimum air temperature was significantly higher in 2024 during February (*p* < 0.001) and October (*p* < 0.05) (Fig. 1c).

**Fig. 2:**
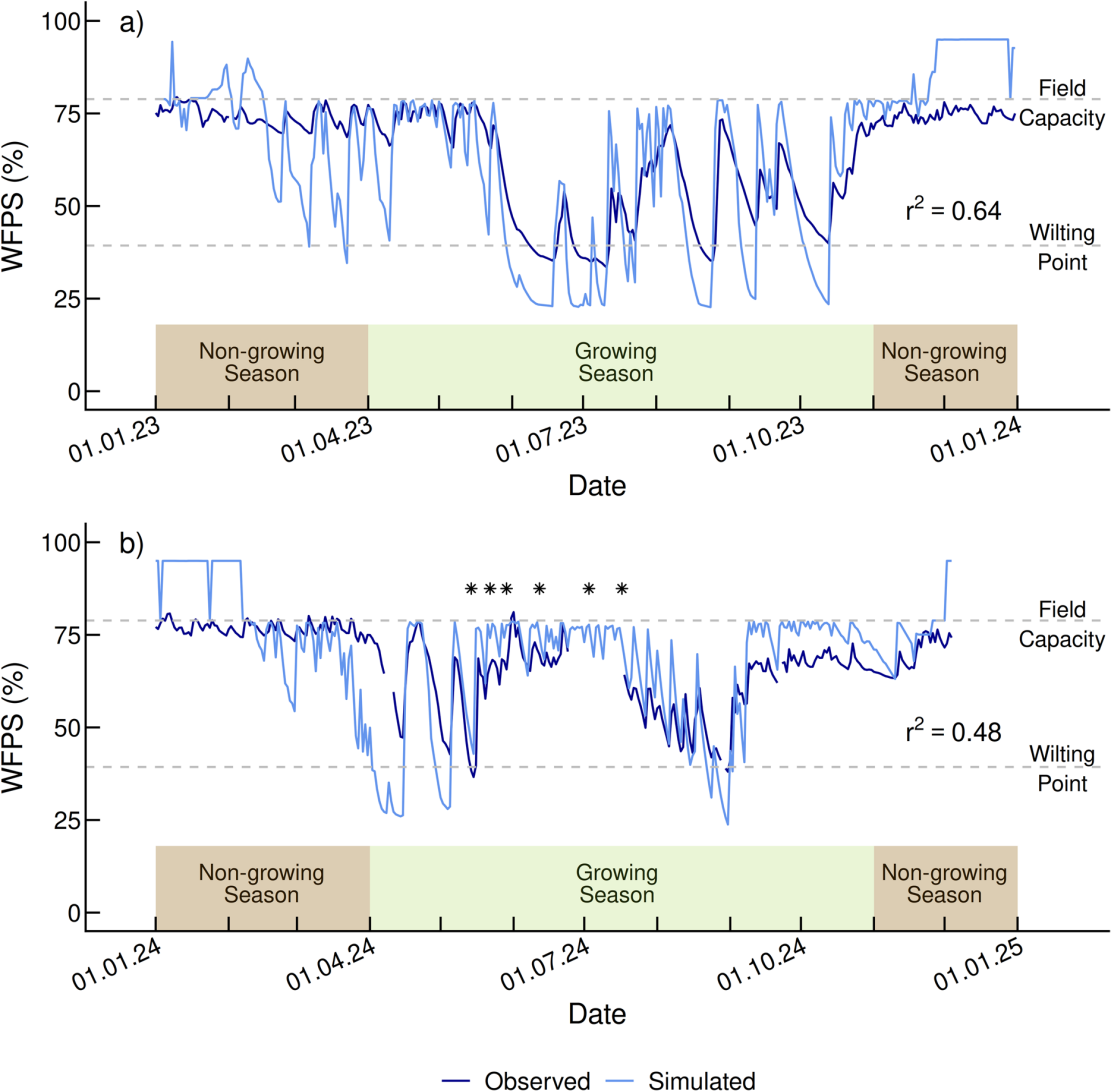
Water-filled pore space (WFPS) measurements (5 cm) throughout 2023 and 2024 at the investigated NPK treatment plot of the Demo site in Switzerland. The observed (dark blue lines) and simulated WFPS values (light blue lines) during the growing season (April–October) and non-growing season (January–March and November–December) are shown. The dashed grey lines indicate the field capacity (78.87%) and wilting point (39.33%). The stars indicate the dates for which the source contributions of N_2_O emissions from the mineral-fertilized sugar beet plot were estimated in 2024

The correlation between the observed and simulated WFPS was moderate in 2023 (r^2^ = 0.64; Fig. 2a) and poor in 2024 (r^2^ = 0.48; Fig. 2b) and common trends from both years highlighted several inconsistencies between the measured and simulated soil water environment. For instance, the range of the simulated WFPS (71) is larger than the measured values (45). Furthermore, a discrepancy between the measured and simulated WFPS occurred between January and mid-February 2023, and again in December 2023 when DayCent overestimated when the soil water content was at or above field capacity. During these periods, the model predicted that the WFPS was at or above field capacity at the 5 cm soil depth (i.e., at or above upper dashed line) 65 days or roughly 18% of the days throughout the year, while the measured observations show that the soil experienced field capacity only 17 days (5%) during the year (Fig. 2). The model’s tendency to overestimate WFPS above field capacity at this site was also seen from January to early February 2024, and in the limited data from December 2024. DayCent simulations throughout 2024 indicated WFPS at or above field capacity for 38 days (10%), whereas actual measurements revealed the soil reached field capacity for only 14 days (4%). During 2023 and 2024 DayCent also estimated that the soil water content was at or below the site’s wilting point more frequently than was measured on the field (i.e., at or below the lower dashed line). DayCent simulated drier soil conditions 55 more days at 5 cm than found in the measured observations in 2023, and 22 more days in 2024. This discrepancy happened at different times each year, but it was most prominent during the growing season. Specifically, DayCent consistently simulated WFPS below the site’s wilting point at various points, including mid-June through mid-July, late August, early September, and late October in 2023, and early to mid-April, early May, and late August in 2024.

### *Fluxes and sources of N*_*2*_*O emissions*

**Fig. 3:**
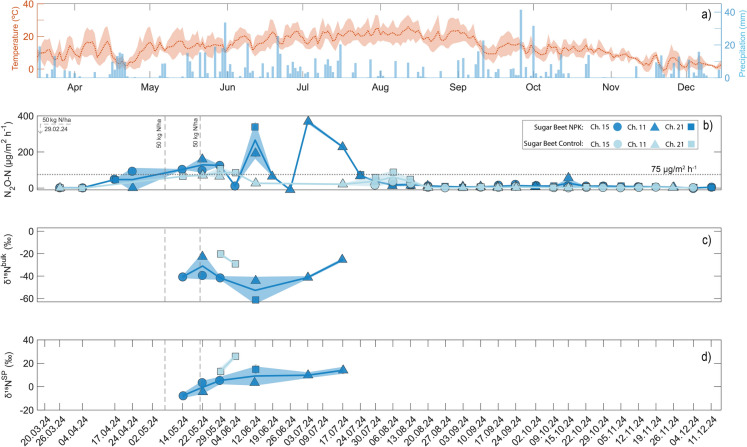
Seasonal course of N_₂_O fluxes and isotopic composition for sugar beet under NPK fertilization vs. control of the Demo experiment: **a** daily air temperature (mean with minimum–maximum range) and precipitation (daily totals), **b** N₂O flux (µg N_2_O-N m⁻^2^ h⁻^1^), **c** δ^18^O, δ^15^N^bulk^ and **d** site preference (δ^15^N^SP^). The stippled line in the N_2_O flux graph represents the 75 µg m^−2^ h^−1^ threshold for isotope flux analysis. Measurement results from different chambers are indicated by specific symbols and colors (dark blue for NPK, light blue for Null treatment)

**Fig. 4:**
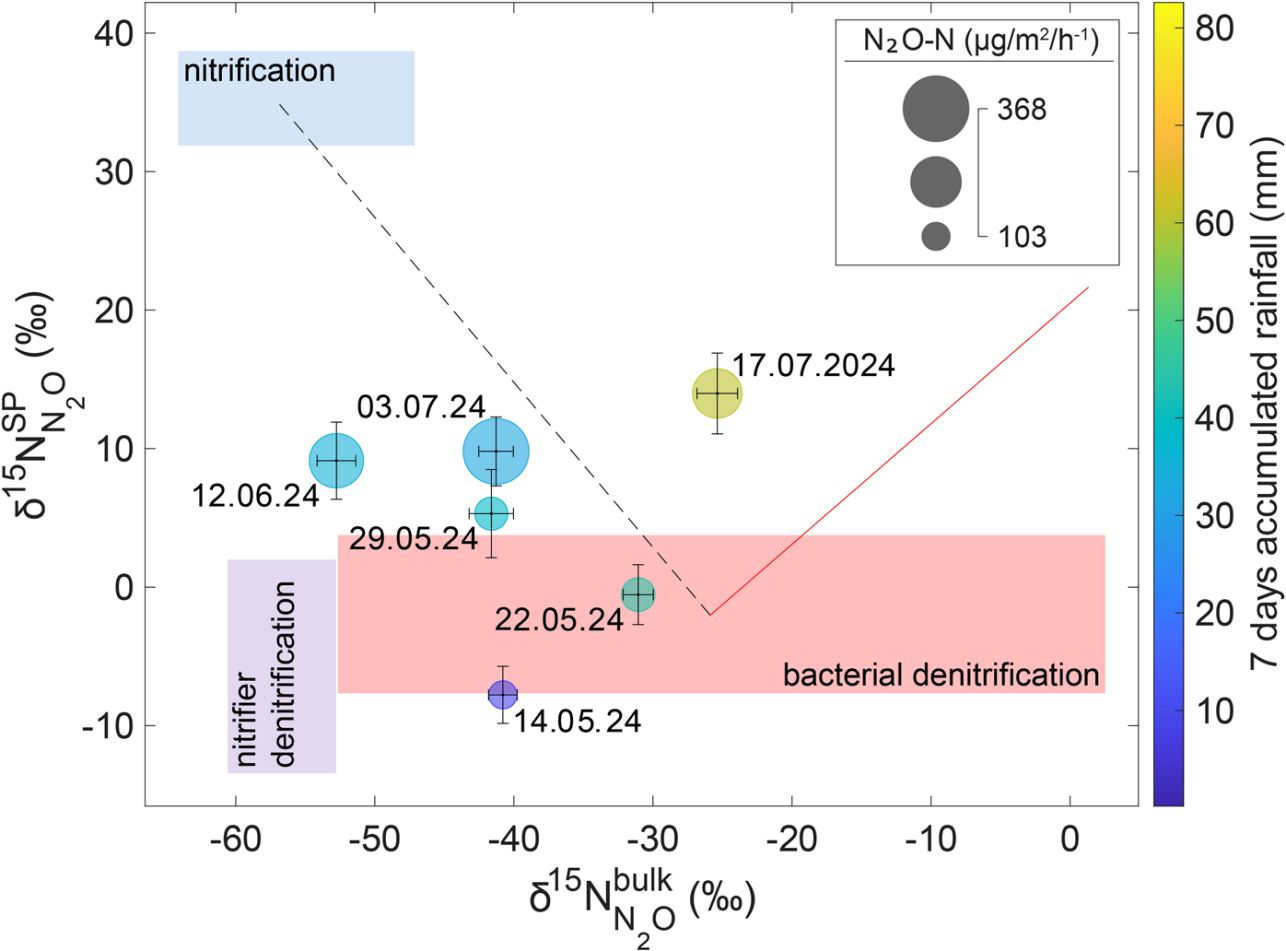
Dual isotope plot of site preference $\delta^{15} N_{{(N_{2} O)}}^{Sp} = (\delta^{15} N^{\alpha } - \delta^{15} N^{\beta } )$ versus $\delta^{15} N_{{(N_{2} O)}}^{bulk} = (\delta^{15} N^{\alpha } + \delta^{15} N^{\beta } )/2$ for N_2_O emitted from the NPK-fertilized sugar beet plot. The points are colour-coded by 7-day accumulated rainfall (mm) and scaled by N₂O flux (µg N/m^2^ h⁻^1^). The coloured boxes indicate ranges of isotopic signatures reported for prominent microbial N_2_O production pathways in literature (Yu et al. 2020). The theoretical N_2_O reduction line (red line) represents isotopic enrichment during N_2_O reduction to N_2_ during denitrification

Across the sampling period (05.03.2024 to 11.12.2024), the integrated weekly average N_2_O flux was generally small and typically below the 75 µg N_2_O-N m^−2^ h^−1^ threshold required for isotopic source member determination (Fig. 3b, dashed line). For the NPK treatment, two larger emission pulses occurred in late spring to mid-summer peaking at ~ 350–400 µg N_2_O-N m^−2^ h^−1^ (Fig. 3b). The emission pulses coincided with periods of increased precipitation and followed the two fertilization events (50 kg N ha^−1^ each) applied at the beginning and the end of May (07.05.2024 and 21.05.2024), expressed by the vertical grey stippled lines in Fig. 3. Isotopic source signatures were only measurable during the high flux periods, while the remaining dataset did not offer sufficiently high N_2_O fluxes to pass the quality criteria and threshold values (${R}_{{[N}_{2}O]}^{2}\hspace{0.17em}$> 0.7, ${flux}_{{[N}_{2}O]}\hspace{0.17em}$> 75 µg N_2_O-N m^−2^ h^−1^ and Keeling line within ± 8 ‰ of ambient air endmember). The cumulative N_2_O emissions observed during this time in the NPK fertilized sugar beet plot with NPK treatment was approximately 79.7% of the annual emissions. For periods with sufficiently high fluxes for isotopic analysis, the dataset indicated that bacterial denitrification is the predominant pathway of N_2_O production in the soils at the Demo site, with most source signatures clustering within or close to δ^15^N^SP^ values reported for denitrification in literature (Yu et al. 2020). This is visualized by the majority of the determined source signatures falling close to or within the red box, signifying denitrification in Fig. 4. To contextualize these findings, the color gradient in Fig. 4 encodes the seven-day accumulated rainfall prior to sampling, while the symbol size scales with the weekly mean N_2_O flux, thereby linking the isotopic source signatures to both the magnitude of the emissions and precipitation. Across the period characterized by largest emissions in late spring to mid-summer (May–July 2024), δ^15^N^SP^ and δ^15^N^bulk^ showed an overall increasing trend, a pattern most consistent with a progressive share of N_2_O reduced to N_2_ via denitrification or, alternatively, an increasing contribution of nitrification (i.e., see dates next to round symbols in Fig. 4). This is most notable during the week with the highest seven-day accumulated precipitation (17.07.2024), where both δ^15^N^SP^ and δ^15^N^bulk^ are shifted towards higher values (Fig. 4). To get a quantitative estimate of process contributions, a Monte Carlo simulation was performed using the FRAME model (Lewicki et al. 2022), which accounts for isotopic fractionation during partial N_2_O reduction. Likewise, the majority of N_2_O emissions for the high flux period was attributed to denitrification (79–96%), whereas nitrification contributed only 4–21% (Table 2). Temporal trends in model results display an increasing share of nitrification derived N_2_O from mid-May to early-July in parallel with drier and better aerated conditions (Table 2, Fig. 3).

**Table 2:** Estimated source contributions of N_2_O emissions from the mineral fertilized sugar beet plot (mean and 95% CI) for bacterial denitrification (bD) + nitrifier denitrification (nD) and nitrification (Ni)

Date	N_2_O flux (µg N_2_O-N m^−2^ h^−1^) ± SE	Fraction of bD + nD	Fraction of Ni
14.05.2024	103 ± 2	0.96 [0.26–1.68]	0.04 [0.001–0.13]
22.05.2024	128 ± 43	0.88 [0.17–1.65]	0.12 [0.004–0.29]
29.05.2024	125 ± 3	0.84 [0.07–1.74]	0.16 [0.005–0.39]
12.06.2024	265 ± 103	0.84 [0.07–1.73]	0.16 [0.010–0.36]
03.07.2024	368 ± 11	0.79 [0.07–1.64]	0.21 [0.014–0.45]
17.07.2024	227 ± 6	0.86 [0.13–1.64]	0.14 [0.004–0.35]

Results include a correction for isotopic enrichment due to microbial N_2_O reduction. Sources bD (bacterial denitrification) and nD (nitrifier denitrification) are combined

Flux values represent the mean across replicate chambers and analyzers, while the uncertainty (SE), incorporates errors of both the regression fit error and variation among chambers

### *N*_*2*_*O emissions and DayCent simulations*

**Fig. 5:**
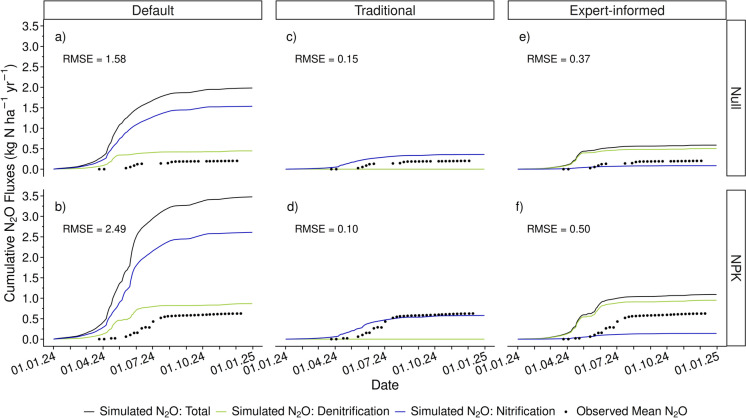
DayCent simulations of the cumulative N_2_O fluxes for the Null and NPK treatments of the sugar beet crop at the Demo site in Switzerland using the default **a**, **b**, traditional calibration **c**, **d**, and expert-informed **e**, **f** parameter values. Total cumulative N_2_O emission (black) and N_2_O production from denitrification (green) and nitrification (blue) are represented by the solid lines, and the observed mean cumulative N_2_O flux measurements are represented by the black dots. The root mean square error (RMSE) represents the deviation between simulated and measured cumulative total N_2_O flux values

The measured, cumulative total N_2_O emission for the NPK fertilizer treatment (0.63 kg N ha^−1^ yr^−1^) at the Demo site was three times higher than that of the Null treatment (0.20 kg N ha^−1^ yr^−1^; Fig. 5). Total N_2_O emissions increased throughout the growing season, together with increased precipitation and fertilization events, between spring and early autumn. DayCent simulations with the default parameter values indicated N_2_O was emitted from the nitrification and denitrification processes, but the total cumulative N_2_O emissions for both the Null and NPK treatments were strongly overestimated (RMSE = 1.58 kg N ha^−1^ yr^−1^ for the Null treatment and 2.49 kg N ha^−1^ yr^−1^ for the NPK treatment) (Fig. 5 a and b). Model fit improved for simulations that were performed with parameter values from the traditional calibration (RMSE = 0.15 for the Null treatment and RMSE = 0.10 kg N ha^−1^ yr^−1^ for the NPK treatment, Fig. 5c, d). However, these simulations attributed all N_2_O emissions to the nitrification process, which is not in line with isotope measurements. This shift to nitrification being the primary N_2_O emission process led to a decrease in N_2_ emissions for the traditional approach when compared to the default approach (from 4.13 to 0.02 kg N ha^−1^ yr^−1^ for the Null treatment and 6.18 kg N ha^−1^ yr^−1^ to 0.03 kg N ha^−1^ yr^−1^ for the NPK treatment) (Fig. S1). The RMSE was slightly greater for the simulations that used the expert-informed parameter values (RMSE = 0.37 kg N ha^−1^ yr^−1^ for the Null treatment and RMSE = 0.50 kg N ha^−1^ yr^−1^ for the NPK treatment, Fig. 5e, f), and these simulations improved estimations of the cumulative total N_2_O flux by limiting N_2_O emissions from nitrification and allocating most of the N_2_O emissions to denitrification in agreement with isotope measurement. N_2_ emissions from the expert-informed approach (4.13 kg N ha^−1^ yr^−1^ for the Null treatment and 6.12 kg N ha^−1^ yr^−1^ for the NPK treatment) corresponded with the default approach (Fig. S1).

## Discussion

### Comparison of measured and modeled WFPS

This study focuses on the parameters that influence the effect of soil water content on the nitrification and denitrification processes in the DayCent model because there were noted discrepancies in the simulated and measured soil water content at this site. These inconsistencies were unaddressed by the model’s default settings and unaccounted for by the traditional calibration. Consequently, predictions of total N_2_O flux using the default settings had the highest error, and predictions of N_2_O contributions from nitrification and denitrification were wrong as compared to source attribution by isotope measurements. While the traditional calibration provided the best model fit for total N_2_O flux, the expert-informed approach better represented N_2_O emissions from nitrification and denitrification at the Demo trial.

Inconsistencies in DayCent’s simulations of soil water content have been observed in other studies (Brilli et al. 2017; Gaillard et al. 2018; Guest et al. 2017; Stehfest and Müller 2004). Overall, factors such as errors in the site input data and limitations in the modeling of soil processes that may impact water flow may account for these variabilities (Del Grosso et al. 2019; Scheer et al. 2014; Wagner-Riddle et al. 2017). Modifying general soil properties could potentially improve DayCent’s prediction of soil water content (Smith et al. 2008). However, N_2_O flux for this region has already undergone extensive calibrations, and maintaining the site-specific soil parameters was preferable to reflect actual soil conditions at the Demo site, as altering them could affect other model outputs such as yields, which were well predicted by the regional study (dos Reis Martins et al. 2022). Therefore, only adjustments to parameters that influence the effect of soil water content on the nitrification and denitrification processes in DayCent were performed at the Demo site because they directly addressed discrepancies in the model’s depiction of the soil moisture environment and its impact on key underlying processes that affect N_2_O emissions from nitrification and dentirification.

Unfortunately, a complete dataset of the measured VWC values from 2024 was unavailable for the sugar beet plot because of complications with fieldwork. As daily temperature and precipitation events differ annually, and water flow is simulated on a daily time step within the model, it is not expected that WFPS simulations would be the same for each year (Del Grosso et al. 2011). However, it was important to compare the temperature and precipitation data from 2023 and 2024 to confirm that the weather at the site was consistent. Since the model used the same input data (site details and soil properties) and the monthly rainfall was not significantly different between 2023 and 2024, it was presumed and confirmed by the limited WFPS data from 2024, that DayCent simulated discrepancies in soil moisture levels similarly for both years.

In this study, the model's misrepresentation of the frequency at which WFPS was above field capacity during the non-growing season and under the site’s wilting point during the growing season may appear contradictory, but these discrepancies stem from distinct sources. For example, at this site DayCent’s simulation of WFPS at or above field capacity was prompted by precipitation events which increased the WFPS more within the model than on the field. This indicates insufficient water movement in the model, possibly because DayCent is unable to represent certain soil hydrological processes, such as preferential macropore flow, which is known to improve drainage. In a study of soil water and N dynamics in eastern Canada, Guest et al. (2017) reported similar inconsistencies between DayCent-simulated and observed VWC at different cropland sites during winter and early spring. The observation that DayCent overrepresented the frequency at which WFPS was at or below the site’s wilting point during late spring and summer aligns with established patterns of WFPS simulations during the growing season (Guest et al. 2017; Jarecki et al. 2008; Smith et al. 2008). Lower precipitation and higher crop transpiration rates during this period resulted in DayCent simulating drier conditions than those measured in the field. Despite being a reasonable representation of soil water flow, the model’s one-dimensional simplification, coupled with DayCent’s lack of consideration of factors like lateral flow, limits its accuracy.

### *Potential and limitations of the applied N*_*2*_*O flux and isotope measurement approach*

While analytics for in-situ high-precision N_2_O concentration and flux measurements has become available in recent years, N_2_O isotope analysis at ambient N_2_O concentration levels is still associated with dedicated analytical setups and considerable efforts in data analysis (Harris et al. 2020; Havsteen et al. 2026). In this study, a very sensitive commercial CRDS analyzer was applied on air samples collected from automatic time integrating chambers situated on agricultural soils to assess N_2_O fluxes and their isotopic composition. To enable accurate analysis of source signatures using a mixing model approach a low flux threshold of 75 µg N_2_O–N m^−^2 h^−1^ was set, which limited sampling dates with valid N_2_O source signature information to a 2-month period after fertilizer application mid-May to mid-July. Better data coverage in future studies can be achieved by adapting chamber closure times to flux conditions. Longer closures for low flux periods would result in higher N_2_O concentrations in the later sampling bags and a lower N_2_O flux threshold, thus more data with valid source signatures. Agreement of isotopic composition at ambient N_2_O with published background values was set as additional quality criteria and filtered out most δ^18^O-N_2_O data. This could be related to the applied non-linearity correction, which was most variable for δ^18^O-N_2_O, but the exact reason for this phenomenon is yet unresolved. Interpretations of N_2_O isotopic source signatures from field data using dual isotope mapping approaches (Yu et al. 2020) or the FRAME model (Lewicki et al. 2022) are still in their beginnings. Due to the multiple production pathways involved (Butterbach Bahl et al. 2013) and uncertainties in the use of laboratory data for comparison, disentangling N_2_O source processes by natural abundance isotopes are an underdetermined problem. In addition, the linkage between isotopic dimensions (e.g. δ^15^N^SP^ and δ^15^N^bulk^ in Fig. 4) remains weak to moderate in the dataset, likely because shifts in isotopic source signatures are not only governed by wetting, but also other factors such as sub-weekly variability in labile carbon and NH_4_^+^or NO_3_^−^availability, the extent of N_2_O reduction and mixing of different N_2_O production pathways typical for agricultural soils (Butterbach-Bahl et al. 2013; Yu et al. 2020). These restrictions can be addressed analyzing independent process parameters (WFPS, NO_3_^−^, NH_4_^+^, etc.), collocated ^15^N-, or ^18^O tracer studies (e.g. Well et al. 2019, Huang et al. 2023) or additional isotopic dimensions (Kantnerova et al. 2022).

### The impact of parameterization on model performance

Inconsistencies in DayCent’s soil water content most likely led to overestimations of N_2_O production from nitrification in the simulations that were performed using the parameter values from the default model settings and the traditional calibration. Within the model, nitrification is the dominant source of N_2_O emissions under aerobic conditions (WFPS between 35 and 60%) (Bateman and Baggs 2005), and N_2_O can also be emitted via nitrification at field capacity depending on the value of the N2Oadjust_fc parameter (as higher values permit increased emissions). Denitrification is regarded as the main pathway that contributes to N_2_O emissions under high soil moisture conditions, which is defined by a specific threshold (WFPS > 60%) in DayCent (Wang et al. 2021). However, most of the N_2_O emissions for both the Null and NPK treatments occurred between April and August, which suggests that denitrification occurs across a broad spectrum of soil moisture conditions. Modifying the N2Oadjust_fc, N2Oadjust_wp, and wfpsdnitadj parameters was required to reconcile the inconsistencies between DayCent’s simulations of WFPS and process-based understanding gathered from the observational methods.

The N2Oadjust_fc and N2Oadjust_wp parameters were largest with the model’s default settings, which led to a considerable overestimation of N_2_O emissions via nitrification and the highest RMSE for this parameterization compared to the two other approaches. While the parameterization of the traditional calibration did not enable N_2_O emissions from nitrification at field capacity, its N2Oadjust_wp value may have still been too high. The majority of N_2_O was produced during the growing season, which is also when the WFPS was simulated but not observed to be below the site’s wilting point. The isotopic composition results showed that although denitrification was the primary source of N_2_O emissions, emissions via nitrification made up for 4–21% of the source contributions throughout the growing season. Setting the N2Oadjust_fc and N2Oadjust_wp parameters to 0.001 better reflected the isotopic composition data, as it allowed for N_2_O emissions from nitrification at field capacity and wilting point without causing a gross overestimation of the total N_2_O flux.

The default setting for the wfpsdnitadj parameter was adequate for the expert-informed approach because it properly reflected the denitrification potential along the soil moisture gradient at the Demo site. Specifically, the wfpsdnitadj parameter value of one allowed denitrification to occur across a wide range of soil moisture conditions. At the Demo site, DayCent simulations using the traditional parameterization inaccurately depicted N_2_O emissions from denitrification because the wfpsdnitadj value of 1.4 restricted such emissions to only occur when the simulated WFPS was at/above the site’s field capacity. As demonstrated in the simulations that used the default and expert-informed parameterizations, a reduction in the wfpsdnitadj parameter resulted in a lower threshold for denitrication, which enabled it to occur under drier soil conditions within the model (Xing et al. 2023). This is supported by the similar cumulative N_2_ emissions from denitrification observed from the default and expert-informed approaches. The traditional approach, however, resulted in smaller, albeit not negligable, N_2_ emissions because denitrification was only permitted under very restricted soil conditions.

## Conclusion

The coupling of N_2_O isotopic source signatures and WFPS measurements with DayCent provided crucial insights into model function through the identification and estimation of key model parameters that influence the impact of soil water content on N_2_O production from nitrification and denitrification. It is important to note that in this study model performance was calibrated and assessed using total N_2_O flux. While measurements of N_2_O isotopic composition identified bacterial denitrification as the predominant source of N_2_O emissions at the Demo site, this may not be the same at other sites. Therefore, this specific expert-informed approach is limited in its site-specific application. However, this analysis demonstrated how a locally adapted biogeochemical model can be used to improve the accuracy of quantifications, which may contribute to the development of small-scale mitigation efforts. Estimating N_2_O emissions from soils is time- and labor-intensive, therefore it is important to have well calibrated models that accurately predict total emissions and the relative contributions of the nitrification and denitrification processes. Thus, the inclusion of other established and emerging observational data may provide additional opportunities to inform model improvement.

## Supplementary Information

Below is the link to the electronic supplementary material.

Supplementary file1 (TIFF 746 kb)

## Data Availability

All data supporting the findings of this study are available within the paper. The observed N2O flux and isotope data will soon be available on the ICOS portal (ICOS Carbon Portal | ICOS).
