# Supplementary figures and images for: Coupling observational methods and the DayCent model to improve examinations of N2O production pathways
Source: Nutr Cycl Agroecosyst. 2026 May 20;133(1):6. doi: 10.1007/s10705-026-10504-1 (PMC13190764; doi:10.1007/s10705-026-10504-1)

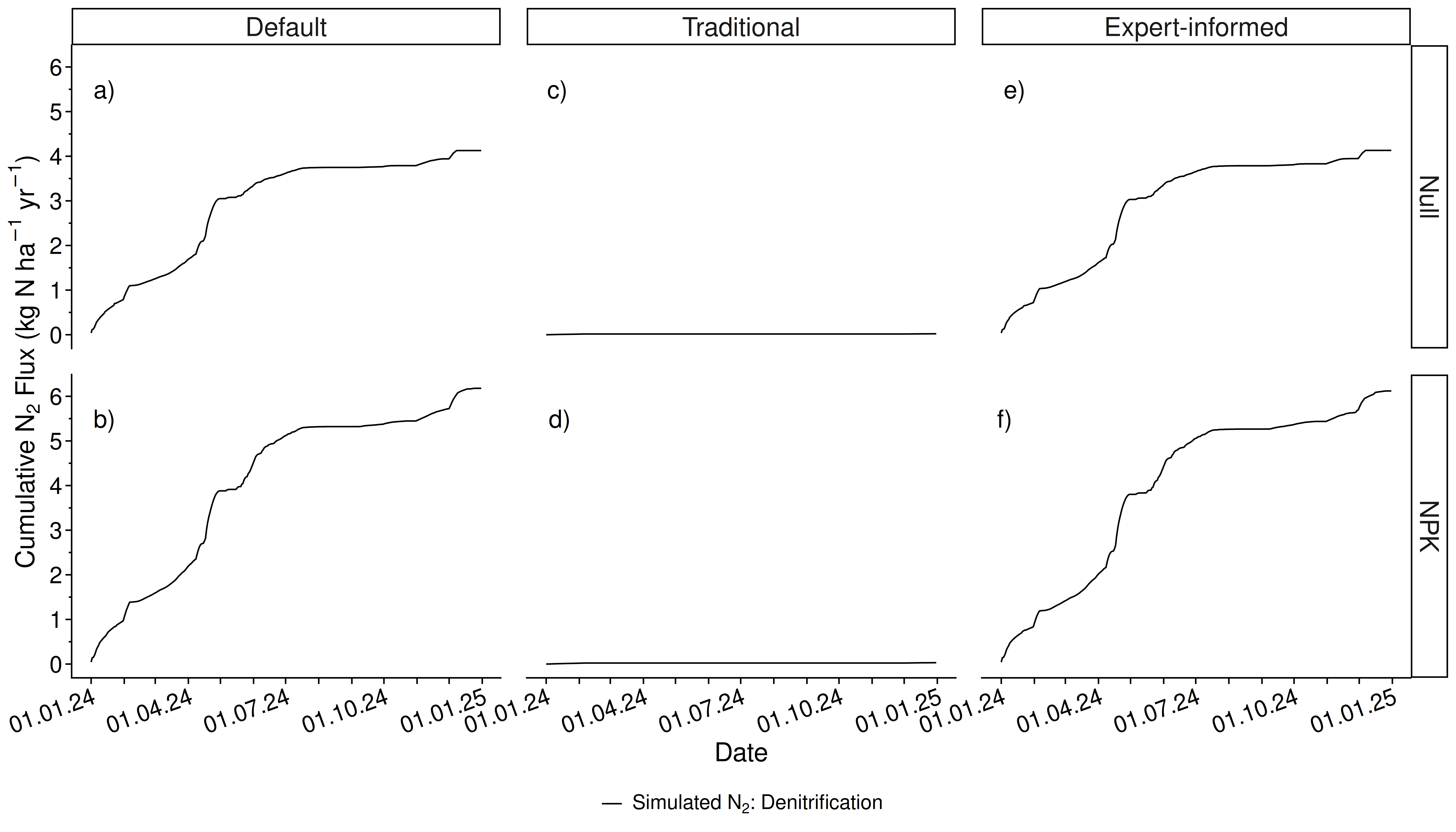

Supplement: Supplementary file 1 — Supplementary file1 (TIFF 746 kb) [file 10705_2026_10504_MOESM1_ESM.tiff]
